# Value Realization and Optimization Path of Forest Ecological Products—Case Study from Zhejiang Province, China

**DOI:** 10.3390/ijerph19127538

**Published:** 2022-06-20

**Authors:** Ning Wang, Caiyao Xu, Fanbin Kong

**Affiliations:** 1 Institute of Ecological Civilization, Zhejiang A&F University, Hangzhou 311300, China; wn1437139016@163.com; 2 Research Academy or Rural Revitalization of Zhejiang Province, Zhejiang A&F University, Hangzhou 311300, China; 3 College of Economics and Management, Zhejiang A&F University, Hangzhou 311300, China

**Keywords:** forest, ecological products, conversion efficiency, super-SBM model, Malmquist

## Abstract

In the last decade, more and more attention has been paid to the efficiency of ecological products’ value in the literature. Studying the value-conversion efficiency of forest ecological products can measure and reflect the huge value contained in forests, which is of great significance to promote the transformation between “clear water and green mountains” and “gold and silver mountains” as well as solve the problem of economic development and environmental protection. Studying the value-conversion efficiency of forest ecological products can scientifically evaluate the results of the mutual transformation of “clear water and green mountains” and “gold and silver mountains”, which is of great significance for deepening the theory of the “two mountains”. This paper took Zhejiang Province as the research object, constructed an index system of forest ecological products’ value accounting, used the super-SBM model and Malmquist index to calculate the conversion efficiency of forest ecological products’ value, and proposed optimization paths according to the research results. The results showed that: (1) From 2000 to 2020, the value of forest ecological products in Zhejiang Province showed a fluctuating upward trend. In 2020, the total value of forest ecological products was RMB 973.717 billion. Among them, the value of material products was RMB 12.560 billion, the value of ecological regulatory products was RMB 726.323 billion, and the value of cultural service products was RMB 234.834 billion. (2) There were great differences in the value-conversion efficiency of forest ecological products among cities in Zhejiang Province, but the overall trend was steady and developing in a positive direction. (3) The total-factor productivity of forest ecological products in Zhejiang Province showed a fluctuating trend, and its growth was mainly limited by the technical efficiency and technological progress index. (4) The main reasons for the conversion-efficiency loss of forest ecological products’ value in Zhejiang Province were excessive input and insufficient output. The specific reasons for the loss of efficiency in different cities were different, so it is necessary to find improvement paths according to local conditions. Our research provides a new perspective for the academic community to evaluate the value-realization effect of ecological products as well as a decision-making reference for policy makers of ecological environmental protection and construction.

## 1. Introduction

Since they were reported in the 1980s, ecosystem services have been attracting a lot of interest. Ecosystem services can be defined as the natural environmental conditions and utility that are formed and maintained by ecosystems for human survival. They provide human beings with food, medicine, and raw materials for industrial and agricultural production, while maintaining human survival and the development of life-support systems [1,2]. The assessment of ecosystem services can be traced back to 1997 [3], and so far the research on assessing the economic value of ecosystem services has covered different ecosystems in different countries and regions [4,5,6,7,8,9]. It can be seen that the evaluation and accounting of the value of ecosystem services have become a hot topic in the fields of economics, ecology, environmental and natural-resource economics, and geography.

Ecological products are in line with ecosystem services, but the difference is that Chinese characteristics are integrated into ecological products. Ecological products are proposed in the context of ecological-civilization construction, which refers to the final products or services that the ecosystem provides for human well-being through biological production and its interaction with human labor, under the conditions of maintaining ecological security and ensuring ecological adjustment functions to create a good life for human beings [10,11,12,13,14]. At present, previously published studies of ecological products’ value mainly focused on theoretical research, mostly for policy analysis and theoretical sorting [15,16,17], and few scholars paid attention to value accounting [18,19,20,21,22,23,24]. The conversion of the value of ecological products was only reflected in the calculation of the Green Gold Index [25,26,27]. To sum up, it can be seen that a number of studies have begun to explore the realization of the value of ecological products. However, the conversion efficiency of ecological products’ value has not been fully explored.

As the most important terrestrial ecosystem on Earth, the forest is the main provider of ecological products. In the forest ecosystem, the interaction of various components allows the forest to possess the functions of water retention, nutrient accumulation, carbon sequestration and oxygen release, air purification, and soil conservation, as well as providing material products and recreational value for human beings [28]. The previous research on forest-ecosystem services was mainly based on theoretical analysis and value evaluation [29,30,31,32,33,34,35,36]. Although extensive research has been carried out on it, few scholars have considered the problem of its value conversion, and no scholar has analyzed it from the perspective of conversion efficiency. How can the conversion efficiency of forest-ecosystem-service value be quantitatively evaluated? This issue is not yet clear. It can provide new ideas for developing the circular economy and solving the problem of resource and ecological-bearing capacity, by researching this aspect [37]. It is also of great significance to promote the comprehensive green transformation of economic and social development.

The conversion efficiency of ecological products’ value refers to the conversion efficiency of gross ecosystem product (GEP) to gross national product (GDP). To the best of our knowledge, there are a lack of papers analyzing conversion efficiency in the literature on the field of ecological products’ value realization, and there exists a gap in this regard. For the mutual conversion between the two, the existing research only stays at the ratio level. However, the ratio method lacks economic significance. The authors have read the literature and found that similar research related to ecological efficiency, energy efficiency, green total-factor productivity (GTFP), and even logistics efficiency are often measured by using SFA, DEA, and DEA-extension methods [38,39,40]. The method incorporates concepts of economic significance such as technology and scale. Therefore, this can provide reference for the research on the value-conversion efficiency of ecological products.

In 2021, the General Office of the Central Committee of the Communist Party of China and the General Office of the State Council issued the “Opinions on Establishing and Improving the Value Realization of Ecological Products”. Thus, it is necessary to promote ecological industrialization and industrial ecologicalization as well as accelerate the improvement of the path to realize the value of ecological products [41]. The national demand for the realization and conversion of ecological products’ value was pointed out. It highlighted the necessity of research on the realization and conversion of ecological products’ value. The realization of the value of ecological products has played a critical role in transforming “clear water and green mountains” into “gold and silver mountains” [42].

In 2017, Zhejiang Province was listed as one of the pilot provinces of the national ecological products’ value-realization mechanism. As a demonstration area for ecological-civilization construction and common prosperity, and also the birthplace of General Secretary Xi Jinping’s ecological-civilization thought, Zhejiang Province took the lead in carrying out the realization of ecological products’ value and formulating the country’s first Gross Ecosystem Product (GEP) standard, “Gross Ecosystem Product (GEP) Accounting Technical Specification for Terrestrial Ecosystems” (DB33/T 2274-2020). Zhejiang Province also has the characteristics of high forest coverage and rich forest resources as well as great potential in the conversion of forest ecological products’ value. Therefore, this paper takes Zhejiang Province as the research object, constructs a forest ecological products’ value-accounting-index system, calculates the value of forest ecological products in Zhejiang Province, and uses the super-SBM model and Malmquist index to study the conversion efficiency of forest ecological products’ value. The aim is to put forward the optimization path of forest ecological products’ value realization in Zhejiang Province. It is expected to provide some references for Zhejiang Province to realize the mutual transformation between “clear water and green mountain” and “gold and silver mountains”, and also provide reference for the accounting of forest ecological products’ value and the evaluation of the conversion effect of “two mountains” in other places in China.

The remainder of this paper is organized as follows. Section 2 introduces the study area, methods, and data sources of this paper. Section 3 describes the model results and summarizes the main findings, while Section 4 discusses and compares them with other studies. Section 5 concludes the full text and suggests future research directions.

## 2. Materials and Methods

### 2.1. Study Area

**Figure 1:**
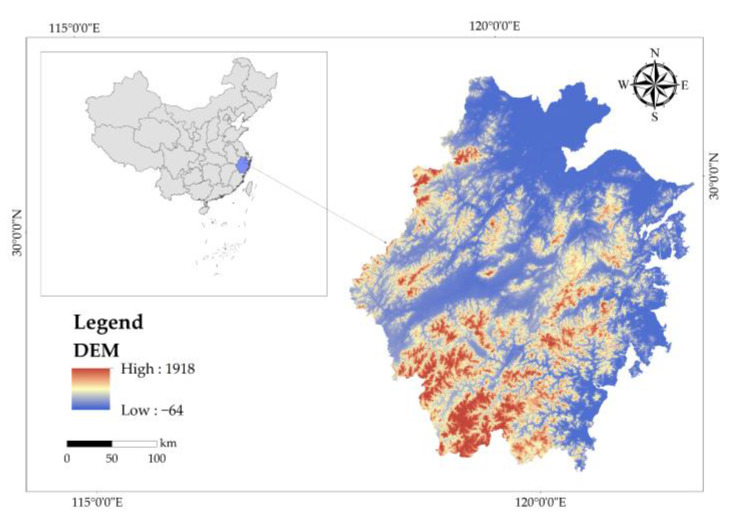
The study area.

Zhejiang Province (27°02′–31°11′ N, 118°01′–123°10′ E) is located in Southeast China, which has a complex geographical situation (Figure 1). Due to its mountainous and hilly terrain, it is known as “seven mountains, one water, and two fields”. Influenced by the subtropical monsoon climate, it has sufficient light and abundant precipitation all around the year, with an average annual precipitation of 1100–2000 mm. It is one of the areas with superior natural conditions in China. According to the data released by the Provincial Forestry Bureau, the existing forest area in the province is 99.03 million acres, and the forest area is 91.13 million acres, of which bamboo forest accounts for about 15.5%, shrub-economic forest accounts for about 13.5%, and arbor forests are the other 71%, with a forest coverage rate of about 61.15%. Zhejiang Province is very rich in forest resources, including 8 major forest types, including coniferous forest, coniferous and broad-leaved mixed forest, evergreen broad-leaved forest, deciduous broad-leaved forest, evergreen and deciduous broad-leaved mixed forest, bamboo forest, economic forest, and mountain dwarf shrub. These combined factors make Zhejiang province appropriate for assessing the value of ecological products.

### 2.2. Data Sources

Data for this study were collected from the 2000–2020 “China Forestry Statistical Yearbook”, “China Urban Statistical Yearbook”, “Zhejiang Statistical Yearbook”, “Zhejiang Natural Resources and Environment Statistical Yearbook”, the “Statistical Yearbook” of each city, Zhejiang Provincial Forestry Bureau, etc. The remote-sensing data for calculating GEP were collected from the Resource and Environmental Science and Data Center of the Chinese Academy of Sciences, the National Qinghai-Tibet Plateau Scientific Data Center, the National Meteorological Data Center, the geographical spatial data cloud, and the National Earth System Science Data Center, while the soil data were from the Chinese soil dataset (1:1 million) in the Harmonized World Soil Database (HWSD), constructed by the Food and Agriculture Organization of the United Nations (FAO) and the International Institute for Applied Systems (IIASA) in Vienna. Among them, since the data for land use are only available for 2000, 2005, 2010, 2013, 2015, 2017, and 2020 (from 2000 to 2020), this paper starts the analysis based on the data of these seven years.

### 2.3. Methods

**Figure 2:**
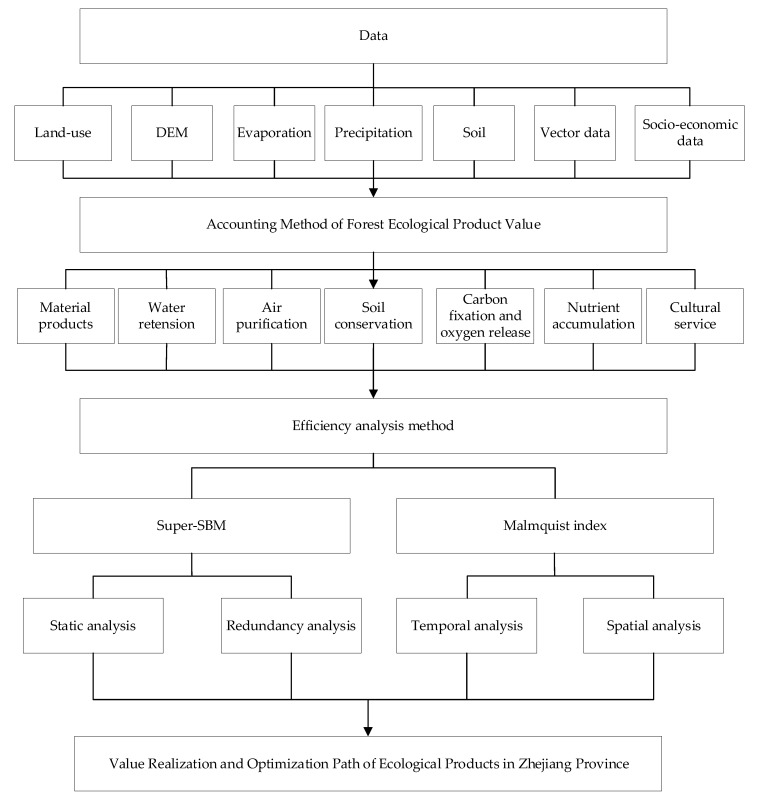
The technology roadmap.

To make the analysis results of the value-conversion efficiency of forest ecological products more scientific and reasonable, this paper selected input and output indicators based on the principles of scientificity, comprehensiveness, and operability. The input indicators included the value of forest ecological products (RMB 100 million), the sum of investments in forestry fixed assets used (RMB 100 million), and the number of employees in the forestry industry (10,000 people). The value of forest ecological products’ accounting referred to the accounting theory and method of ecosystem-service-function value by scholars such as Ouyang Zhiyun et al. [1]. It is represented by the sum of material-product value, regulatory-products value, and cultural-service value. First, the physical quantity was calculated through the InVEST model, and then the value was calculated according to the “Gross Ecosystem Product (GEP) Accounting Technical Specification for Terrestrial Ecosystems”. Since there is no direct data on the number of employees in the forestry industry, they were obtained by indirect calculation. The output value of primary, secondary, and tertiary production of the forestry industry was taken as the output index. Therefore, this paper constructed the conversion-efficiency-accounting-index system of forest ecological products’ value, as shown in Table 1. In addition, the research was carried out according to the technical roadmap shown in Figure 2.

#### 2.3.1. Value-Accounting Method of Forest Ecological Products

The value of forest ecological products should be calculated according to the following formula.

GEP = EPV + ERV + ECV(1)

where GEP represents the value of forest ecological products (RMB/a), EPV is the total value of material products (RMB/a), ERV is the total regulatory products value (RMB/a), and ECV is the total value of cultural service (RMB/a).

##### Accounting of Material-Products Value

The value of material products includes the value of forest products and forest by-products in Zhejiang Province, which are accounted for by the marketing value method.

(2) $$\mathrm{EPV}=\sum_{i=1}^{n}A_{i}{\;\times \;P}_{i}$$

where EPV is the value of material products (RMB/a), $A_{i}$ is the output of class i products (t/a), and $P_{i}$ is the price of class i products (RMB/t).

##### Accounting of Regulatory-Products Value

The value of regulatory products includes water-retention value, nutrient-accumulation value, soil-conservation value, air-purification value, carbon fixation, and oxygen-release value.

(3) $$\mathrm{ERV}={\;V}_{\mathrm{wr}}\;+{\;V}_{\mathrm{an}}\;+{\;V}_{\mathrm{sr}}\;+V_{a}+{\;V}_{\mathrm{cf}}\;+{\;V}_{\mathrm{or}}$$

where ERV is the value of the regulatory products (RMB/a), $V_{\mathrm{wr}}$ is the value of water retention(RMB/a), $V_{\mathrm{an}}$ is the value of nutrient accumulation (RMB/a), $V_{\mathrm{sr}}$ is the value of soil conservation (RMB/a), V_a_ is the value of air purification (RMB/a), $V_{\mathrm{cf}}$ is the carbon fixation value, and $V_{\mathrm{or}}$ is the value of oxygen release (RMB/a).

1. Value of Water Retention

Water retention refers to the remaining part of the runoff lost after the water yield minus the soil water saturation, and the water yield refers to the remaining water on and under the surface of the ground after the precipitation minus the actual evaporation [43]. The calculation formula is:

(4) $$Y_{\left(X\right)}=\left(1\;-\;\frac{{\mathrm{AET}}_{\left(X\right)}}{P_{\left(X\right)}}\right){\;\times \;P}_{\left(X\right)}\frac{{\mathrm{AET}}_{\left(X\right)}}{P_{\left(X\right)}}=1+\frac{{\mathrm{PET}}_{\left(X\right)}}{P_{\left(X\right)}}\;-\;{\left[1+{\left(\frac{{\mathrm{PET}}_{\left(X\right)}}{P_{\left(X\right)}}\right)}^{w}\right]}^{1∕w}$$

(5) $${\mathrm{PET}}_{\left(X\right)}{=K}_{c\left(x\right)}{\;\times \;\mathrm{ET}}_{o\left(x\right)}$$

(6) $$W_{\left(X\right)}=\frac{{\mathrm{AWC}}_{\left(X\right)}\;\times \;Z}{P_{\left(X\right)}}+1.25$$

(7) $$V_{\mathrm{wr}}{=Y}_{\left(X\right)}\;\times \;C\;\times \;\delta$$

where $Y_{\left(X\right)}$ is the annual water output of a landscape type (mm), $\frac{{\mathrm{AET}}_{\left(X\right)}}{P_{\left(X\right)}}$ is the actual annual evapotranspiration of the grid unit (mm), $W_{\left(X\right)}$ is the annual precipitation of the grid unit (mm), ${\mathrm{PET}}_{\left(X\right)}$ is the potential evapotranspiration of grid unit X, $K_{c\left(x\right)}$ is the evapotranspiration coefficient of crops, ${\mathrm{ET}}_{o\left(x\right)}$ is the reference (crop) evapotranspiration, ${\mathrm{AWC}}_{\left(X\right)}$ is the available water content of plants, w is the empirical parameter, Z is Zhang coefficient, C is water price (RMB/m^3^), and δ is premium coefficient (default is 1).

2. Value of Nutrient Accumulation

During the growth process, the forest will continuously absorb N, P, K, and other nutrients and store them. Therefore, nutrient accumulation in the forest ecosystem is mainly reflected by the nutrients (N, P, K) absorbed by trees every year, that is, the accumulation of N, P, and K in the forest ecosystem. According to the indirect value of nutrient cycling in China’s ecosystem calculated by Ouyang Zhiyun, it can be calculated that the ecosystem can accumulate 0.025426 g nitrogen, 0.00201 g phosphorus, and 0.01012 g potassium for every 1 g of fixed carbon [44].

(8) $$V_{\mathrm{an}}\;=(C_{\mathrm{tot}}\;\times \;0.025426+{\;C}_{\mathrm{tot}}\times 0.00201+{\;C}_{\mathrm{tot}}\times 0.01012)\;\times \;P$$

where: $V_{\mathrm{an}}$ is the nutrient accumulation value (RMB/a), $C_{\mathrm{tot}}$ is the total carbon storage, and P is the price of fertilizer (RMB). In 1990, the average price of chemical fertilizers (in scalar quantity) in China was 2549 RMB/t, so the price in 2015 was 5756 RMB/t after accounting for the inflation rate of the accumulated 25 years.

3. Value of Soil Conservation

Soil conservation refers to the difference between the amount of soil erosion that may occur without surface-vegetation coverage and the amount of soil erosion under the current surface-vegetation coverage [45]. The value of soil conservation is accounted for by the alternative-cost method.

(9) SRET=RKLS − USLE

(10) RKLS=R × K × LS

(11) USLE=R × K × LS × C × P

(12) $$V_{\mathrm{sr}}{=\lambda \;\times \;(Q}_{\mathrm{sr}}/\rho )\;\times \;C$$

where SRET is the sediment retention of the block (t), RKLS is the potential soil loss (t), R is the rainfall erosion factor MJ·mm/(hm^2^·h·a), P is the annual rainfall (mm), K is the soil erodible factor, USLE is the actual soil loss (t), C is the vegetation cover and management factor, P is the factor of soil-conservation measures, λ—sedimentation coefficient, ρ—soil bulk density (t/m^3^), and C is the cost of desilting works per unit storage capacity of the reservoir (RMB/m^3^).

4. Value of Air Purification

It is the function of the natural ecosystem to absorb, filter, decompose, and reduce atmospheric pollutants, thereby effectively purifying the air and improving the atmospheric environment. The air-pollutant-purification amount is used as the accounting index, and the calculation is performed by the alternative cost method in accordance with the provisions of GB3095-2012, for the items that should be controlled for ambient air quality.

(13) $$Q_{\mathrm{ap}}\;=\sum_{i=1}^{n}Q_{i}$$

(14) $$V_{a}\;=\sum_{i=1}^{n}Q_{\mathrm{api}}\;\times \;C_{i}\;$$

where $Q_{\mathrm{ap}}$ represents the total emission of air pollutants (kg/a), $Q_{i}$ is the emission of class i air pollutants (kg/a), i is the category of pollutants, i = 1, 2, …, n, dimensionless, n is the number of air-pollutant categories without dimension, $Q_{\mathrm{api}}$ is the purification amount of the I air pollutant (t/a), and $C_{i}$ is the treatment cost of class i air pollutants (RMB/t).

5. Value of Carbon Fixation and Oxygen Release

It is the function of natural ecosystems to absorb carbon dioxide in the atmosphere through plant photosynthesis, synthesize organic matter, release oxygen, fix carbon in plants or soil, maintain the stability of atmospheric oxygen, and regulate atmospheric components [21].

The calculation formula of carbon fixation value is:

(15) $$C_{\mathrm{tot}}\;={\;C}_{\mathrm{above}}{+C}_{\mathrm{below}}{+C}_{\mathrm{soil}}{+C}_{\mathrm{dead}}\;$$

(16) $$V_{\mathrm{cf}}{=C}_{\mathrm{tot}}{\;\times \;P}_{{\mathrm{co}}_{2}}$$

where $C_{\mathrm{tot}}$ represents the total carbon storage, $C_{\mathrm{above}}$ refers to aboveground biological carbon storage, $C_{\mathrm{below}}$ is the underground biological carbon storage, $C_{\mathrm{soil}}$ is soil carbon storage, $C_{\mathrm{dead}}$ is the carbon storage of litter, and $P_{{\mathrm{co}}_{2}}$ is the carbon-trading price (RMB/t).

The calculation formula of the oxygen-release value is:

(17) $$V_{\mathrm{or}}{=Q}_{\mathrm{or}}{\;\times \;P}_{O_{2}}$$

where $Q_{\mathrm{or}}$ is oxygen release (t), and $P_{O_{2}}$ is the price of oxygen release (RMB/t).

##### Accounting of Cultural-Service Value

The value of cultural service is replaced by the income of forest tourism in Zhejiang Province. We assume that forest tourism accounts for 30% of total income [25].

#### 2.3.2. Super-SBM Model

Data Envelopment Analysis (DEA) can evaluate the relative efficiency of multiple decision-making units (DMUs), each of which has multiple inputs and multiple outputs [39]. The traditional DEA model is generally analyzed from the radial angle, and the relaxation variables are not included in the analytical framework. Moreover, multiple results are at the forefront at the same time, and the calculated efficiency value is high. In this regard, Tone first proposed the SBM model and then introduced the super efficiency SBM model, namely the super-SBM model [46], based on SBM, which also solved the problem of distinguishing multiple efficient decision-making units. It can effectively measure the relationship between input and output. Based on the establishment of the non-oriented Super-SBM model, this paper regards each city as a decision-making unit (DMU), and uses DEA SolverPro 5.0 software to measure the value-conversion efficiency of the ecological products in cities in Zhejiang from 2000 to 2020. The specific-model-construction method of the super-SBM model is as follows [47,48].

(18) $$\min\theta =\frac{1+\frac{1}{m}\sum_{i=1}^{m}\frac{S_{i}^{-}}{x_{\mathrm{ik}}}}{1-\;\frac{1}{S}(\sum_{r=1}^{S}\frac{S_{t}^{+}}{y_{\mathrm{tk}}}\;)}$$

s. t.

(19) $$\sum_{j=1,\;j\neq k}^{m}x_{\mathrm{ij}}\lambda_{j}\;{-\;S}_{i\;}^{-}\leq {\;x}_{\mathrm{ik}}\;(i=1,2,\ldots ,\;m)$$

(20) $$\sum_{j=1,\;j\neq k}^{m}y_{\mathrm{tj}}\lambda_{j}{+S}_{t}^{+}\;\geq {\;y}_{k}\;(t=1,2,\ldots ,\;s)$$

where θ is the relative efficiency value of the decision-making unit; $\lambda_{j}\;\geq \;0,\;j=1,\;2,\;\ldots ,\;n\;(j\;\neq \;k),{\;S}_{i}^{-}\;\geq {\;0,\;\mathrm{and}\;S}_{t}^{+}\;\geq \;0.\;x,\;y$ represent input and output variables; m and s represent the number of input and output indicators, and $S_{i}^{-}\;\mathrm{and}\;S_{t}^{+}$ represent the slack variables of input and output, respectively. $\lambda_{j}$ is the weight variable.

#### 2.3.3. Malmquist Index

The super-SBM model can effectively evaluate the cross-sectional data of value-conversion efficiency of ecological products in cities. However, the realization of ecological products’ value itself is a dynamic process, and the external environment of the decision-making unit will change with time. Total-factor productivity is a dynamic measure. Therefore, the Malmquist index was used to analyze the dynamic changes in the value-conversion efficiency of forest ecological products with the help of DEAP 2.1 software [49,50].

(21) $${M(x}_{t+1}{,\;y}_{t+1}{;\;x}_{t}{,\;y}_{t})={\left[\frac{D_{i}^{t}{(x}_{t+1}{,\;y}_{t+1})}{D_{i}^{t}{(x}_{t}{,\;y}_{t})}\times \frac{D_{i}^{t+1}{(x}_{t+1}{,\;y}_{t+1})}{D_{i}^{t+1}{(x}_{t}{,\;y}_{t})}\right]}^{\frac{1}{2}}$$

where $x_{t}$ and $x_{t+1}$ represent the input index vectors of the t and t + 1 periods, respectively, $y^{t}$ and $y^{t+1}$ represent the output index vectors of the t and t + 1 periods, respectively, and $D_{i}^{m}{(x}^{m}{,y}^{m})$ represents the input distance function of the input generation efficiency in phase t and phase t + 1, respectively (m = t, t + 1).

Further, under the assumption of constant return to scale, the Malmquist index can be decomposed into the technical-efficiency-change index (Effch) and technological-progress index (Techch).

(22) $$\mathrm{Effch}=\frac{D_{i}^{t+1}{(x}_{t+1}{,\;y}_{t+1})}{D_{i}^{t}{(x}_{t}{,\;y}_{t})}$$

(23) $$\mathrm{Techch}={\left[\frac{D_{i}^{t}{(x}_{t+1}{,\;y}_{t+1})}{D_{i}^{t}{(x}_{t}{,\;y}_{t})}\times \frac{D_{i}^{t+1}{(x}_{t+1}{,\;y}_{t+1})}{D_{i}^{t+1}{(x}_{t}{,\;y}_{t})}\right]}^{\frac{1}{2}}$$

(24) $${M(x}_{t+1}{,\;y}_{t+1}{;x}_{t}{,\;y}_{t})=\mathrm{Effch}\;\times \;\mathrm{Techch}$$

When the return to scale is variable, the technical-efficiency-change index can be further decomposed into the pure-technical-efficiency index (Pech) and scale-efficiency index (Sech).

(25) $$\mathrm{Pech}=\frac{D_{o}^{t+1}{(x}_{t+1}{,\;y}_{t+1})}{D_{o}^{t}{(x}_{t}{,\;y}_{t})}$$

(26) $$\mathrm{Sech}=\left[\frac{D_{o}^{t}{(x}_{t}{,\;y}_{t})}{D_{o}^{t+1}{(x}_{t+1}{,\;y}_{t+1})}\times \frac{D_{i}^{t+1}{(x}_{t+1}{,\;y}_{t+1})}{D_{i}^{t+1}{(x}_{t}{,\;y}_{t})}\right]\;\times \;{\left[\frac{D_{i}^{t}{(x}_{t}{,\;y}_{t})}{D_{i}^{t+1}{(x}_{t+1}{,\;y}_{t+1})}\times \frac{D_{i}^{t}{(x}_{t+1}{,\;y}_{t+1})}{D_{i}^{t+1}{(x}_{t}{,\;y}_{t})}\right]}^{\frac{1}{2}}$$

(27) $${M(x}_{t+1}{,\;y}_{t+1}{;\;x}_{t}{,\;y}_{t})=\mathrm{Pech}\;\times \;\mathrm{Sech}\;\times \;\mathrm{Techch}$$

## 3. Results

### 3.1. GEP Value-Accounting Results

Table 2 presents that the value of forest ecological products in Zhejiang Province in 2020 was RMB 973.717 billion, which was about 51 times the total output value of forestry. Among them, almost three-fourths of the total value (74.6%) were regulatory products, which had the highest value, of RMB 726.323 billion. The value of cultural service products was RMB 234.834 billion, accounting for 24.12% of the total value, and the value of material products was RMB 12.560 billion, accounting for 1.29%. From the perspective of all ecological products, the overall rank order for each ecological products based on their contributions to overall value was as follows, from high to low: carbon fixation and oxygen-release value, soil-conservation value, cultural-service value, nutrient-accumulation value, material-product value, air-purification value, and water-retention value. The proportions were listed in Table 2. Among them, carbon fixation and oxygen release, soil conservation, and cultural service accounted for the largest proportion, and the combined proportion of the three items was about 95.56% of the total value of forest ecological products.

**Figure 3:**
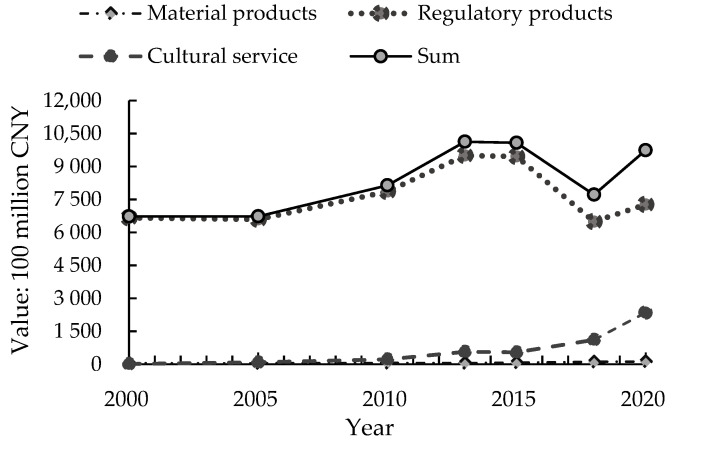
The changing trend of the value of various ecological products in Zhejiang Province from 2000 to 2020.

As shown in Table 3 and Figure 3, from 2000 to 2020, the value of forest ecological products in Zhejiang Province showed a fluctuating upward trend, rising from RMB 671.124 billion in 2000 to RMB 973.717 billion in 2020, an increase of about 45.09%. The peak occurred around 2013–2015. The value of material products, the value of regulatory products, and the value of cultural service all showed an upward trend. In terms of items, in addition to air purification, the regulatory products all showed an upward trend, while the value of air purification showed a decreasing trend. The value of cultural service increased from RMB 1.384 billion in 2000 to RMB 234.834 billion in 2020.

### 3.2. Static Analysis

Table 4 shows the city comparison and differences in the value-conversion efficiency of ecological products in Zhejiang Province from 2000 to 2020. Overall, the value-conversion efficiency of forest ecological products in Zhejiang Province was relatively high. Except for Hangzhou and Wenzhou, the value-conversion efficiency of forest ecological products has reached an effective level. Among the 11 cities, Jiaxing had the highest value-conversion efficiency of forest ecological products, at 5.56, and Hangzhou had the lowest efficiency, at 0.52. The extreme difference of 5.04 indicated that the value-conversion efficiency of forest ecological products in Zhejiang Province varied greatly. It can also be found that although the value-conversion efficiency of forest ecological products in Hangzhou was the lowest, it showed a rapidly rising trend with time. The conversion efficiency of Wenzhou also showed a trend of the annual growth trend. From the data in Table 4, the conversion efficiency of Wenzhou’s forest ecological products has reached 1 since 2015, indicating that it has reached an effective level.

### 3.3. Dynamic Analysis

#### 3.3.1. Time Dimension

As can be seen from Table 5, on the whole, the average total-factor productivity of Zhejiang Province was 1.04, and it increased from 0.006 to 1.419 from 2000 to 2010, showing a rapid-growth trend. However, it started to decline and fluctuated continuously from 2010, dropping to 0.703 in 2020.

In terms of the index composition, the average technical efficiency from 2000 to 2020 was 1.016, and the technological progress rate was 1.024. The technological-progress index from 2000 to 2020 showed large fluctuations, which indicated a trend of synchronous fluctuations with the total-factor growth rate. However, the technical-efficiency-variability index, pure-technical-efficiency-variability index, and scale-efficiency-variability index did not change much.

#### 3.3.2. Spatial Dimension

The Malmquist index and its decomposition results for cities in Zhejiang Province are presented in Table 6. On the whole, the gaps in technical efficiency, technical progress index, pure-technical efficiency, and scale efficiency among cities were relatively small. From the perspective of cities, from 2000 to 2020, the total-factor productivity of five cities in Zhejiang Province exceeded 1 (Table 6), accounting for 45.5%. Among them, Lishui had the highest total-factor productivity, followed by Hangzhou, with an average annual growth rate of 3.1%. Shaoxing had the lowest total-factor productivity, showing a downward trend with an annual average of 28.8%. Wenzhou, Jiaxing, Huzhou, Quzhou, and Taizhou also showed declines, and their total-factor productivity showed an annual average decline of 11.4%, 25.7%, 1.1%, and 20.4%, respectively.

### 3.4. Input–Output Slack-Rate Analysis

The calculation result in Table 4 shows that some cities were in the DEA invalid state every year during the study period, which indicates that these cities had efficiency loss to varying degrees in the realization of the value of forest ecological products. According to the connotation of efficiency, the realization of an effective state requires that the given output be achieved with as little factor input as possible, or the maximum agricultural output can be achieved under the given factor input. The purpose of this part is to put forward improvement paths for improving the value-conversion efficiency of forest ecological products in various cities in Zhejiang Province.

Table 7 presents the annual average regional input and output redundancy and insufficiency in all cities in Zhejiang Province, that is, the slack rate. It can be seen from the above that the efficiency of the nine cities, except Hangzhou and Wenzhou, has reached the frontier of production and achieved effectiveness. So, these nine cities are not within the scope of discussion. As shown in Table 7, it can be found that the output value of the secondary forestry industry in Hangzhou presented a deficit of 2.33%, and several input indicators showed a certain redundancy. Wenzhou had 15.87% redundancy in the number of people in the forestry industry, and a 14.23% shortage in the output value of the secondary forestry industry. In terms of output alone, although Jiaxing and Huzhou ranked higher, there was still a certain gap between the expected output and the optimal value.

## 4. Discussion

From the GEP accounting results, our research showed that the three ecological products of carbon fixation and oxygen release, soil conservation, and cultural service are the main ecological products of forests in Zhejiang Province. It reflects that the forest ecosystem in Zhejiang Province plays an important role in carbon fixation, oxygen release, and soil conservation. The cultural service value was as high as RMB 234.834 billion. There are several possible explanations for this result. One is that it might be related to the fact that Zhejiang Province has many national-forest-tourism demonstration cities, demonstration counties, and many national 5A-level scenic spots. The other is that the high cultural-service value is the result of the rising forest area, which also allows the supply capacity of the forest ecosystems for ecological products to continuously improve. The value of forest ecological products has increased year by year, indicating that the state of the forest ecosystem in Zhejiang Province has been improving continuously, and the forestry industry, especially the forest-tourism industry, has gradually prospered. In particular, researchers [51] estimated the Chinese ecosystem-services value to be 1.73 times the Chinese GDP. This study estimated the total value to be 51 times the total output value of forestry, which means that the level of ecological products’ values in Zhejiang is significantly higher than the national average. The overall value of forest ecological products is very high.

From the perspective of the value-conversion efficiency of forest ecological products:

(1) In terms of static, the overall situation of the value-conversion efficiency of forest ecological products in Zhejiang Province was relatively good. Jiaxing, with the highest efficiency, takes ‘Painting Water Country, Forest Jiaxing’ as the construction concept to comprehensively promote the development of the forestry industry. Its high-conversion efficiency of forest ecological products’ value also indicates that forest input–output structure is better and resources are fully and effectively utilized. Although Hangzhou had the lowest efficiency, its forest-cover rate ranks first among national provincial capitals. Relying on the advantages of forest resources, Hangzhou continues to expand and strengthen traditional industries, such as flowers and seedlings, woody oil, and bamboo. At the same time, it is expanding new formats, such as forest health and green leisure. We believe that the value-conversion efficiency of forest ecological products in Hangzhou will be improved soon.

(2) In terms of dynamics, the total-factor-growth rate of 1.04 showed that the value-conversion efficiency of forest ecological products in Zhejiang Province is on the rise as a whole. From 2000 to 2010, the overall total-factor productivity of Zhejiang Province continued to increase, and though it declined from 2010 to 2020, the technological progress index showed the same fluctuation trend during this period. It suggests that the elevation in total-factor productivity is inseparable from technological progress. The key to total-factor productivity is to promote technological progress. From 2000 to 2010, the output value of the forestry industry showed an upward trend. A likely explanation is that this was the early stage of forestry development. We speculate that a series of human activities made the development and utilization of forest resources larger. However, China began to gradually realize the importance of ecological-civilization construction after 2010, especially since it was included in the report of the 18th National Congress of the Communist Party of China in 2012. Since then, the ecological-civilization construction has been further improved. Many provinces and cities have implemented policies on returning farmland to forest and afforestation subsidies. During this process, the output of wood began to decrease greatly, and the growth rate of the forestry-output value slowed down. However, the continuous improvement of forest coverage also made the value of forest ecological products continue to increase, and the total-factor productivity also gradually increased. From the perspective of cities, there is a high coincidence between the fluctuation trend of total-factor productivity and the change of the technological-progress index in Zhejiang Province, which was similar to the overall situation. In addition, the total-factor productivity of many cities in Zhejiang Province was lower than 1. Therefore, it is necessary to further stabilize forestry development and strengthen technical investment. In terms of sub-indices, the technical-efficiency index of Hangzhou and Ningbo was greater than 1, while the technological-progress index was less than 1. It suggests that the improvement of technical efficiency in these two cities is the main reason that affects the value-conversion efficiency of forest ecological products, while technological progress does not show a positive effect. Therefore, technological progress and its components, including technological changes and technological-efficiency changes, are definitely a powerful, driving force for the realization of the value of forest ecological products in Zhejiang province. Therefore, the government should establish a corresponding forestry technical-service structure, continuously publicize and promote scientific and technological forestry achievements, increase the proportion of technical input in forestry production, and promote scientific and technological forestry progress. According to previous research [52,53], forest-related enterprises can also actively carry out green-technology innovation, which is not only conducive to improving their own financial performance but also conducive to the technological progress of forestry. It should also promote exchanges and cooperation between regions, so that high-efficiency areas play a leading role, become a model for low-efficiency areas, and realize the overall improvement of the conversion efficiency of forest ecological products’ value.

(3) From the perspective of the input–output slack rate, referring to researcher’s practice [54], this paper analyzed the direction in which the value-conversion efficiency of ecological products can be improved, from the perspective of the input–output slack rate. The results (Table 7) indicate that the low resource-utilization rate and insufficient output of the secondary forestry industry are important reasons for the inefficiency in Hangzhou. Only by improving resource utilization, allocating resources rationally, and promoting industrial development can the value-conversion efficiency of forest ecological products be improved. Wenzhou should not only consider promoting industrial development but also consider the rational allocation of elements. It needs to save labor input to improve the total-factor productivity of the forest. The findings from the study suggest that the most important reason for the loss of value-conversion efficiency of forest ecological products in Zhejiang Province is the redundancy of input resources, among which the redundancy of forestry practitioners is the most serious. In addition to transferring surplus forestry labor and reducing personnel input, improving the overall quality of forestry practitioners is the fundamental solution to the problem. These results would seem to suggest that government departments need to pay more attention to the forestry industry and strengthen the professional knowledge of employees by means of education and training. The improvement of the overall education level can not only save human capital but also promote the technological progress of the forestry industry. It can also improve total-factor productivity while promoting forestry development. Due to the difference in the efficiency loss of ecological products in different cities, each city should take appropriate measures to improve the value-conversion efficiency of forest ecological products, according to the actual situation.

It is of great significance to quantify the value of ecological products and scientifically evaluate the transformation effect of “clean water and lush mountains” to “gold and silver mountains”. This study was based on economic theory and methods, combined with the knowledge of geography, to quantitatively calculate the transformation effect of the “two mountains” in Zhejiang Province and identify why those areas were inefficient. At the same time, it revealed the importance of resource allocation to the realization of the value of ecological products. It has an important theoretical value for improving the value of forest ecological products and also provides an important reference for the transformation of the “two mountains” in Zhejiang Province of China. So, the theoretical and practical contributions of this paper are as follows: First, this paper enriches the accounting research of ecological products’ value, discusses the relationship between ecological products’ value and economic value, supplements the theoretical system for evaluating the conversion effect of ecological products’ value, improves the evaluation index system for forest ecological products’ value, and makes the theoretical system more complete. Second, Zhejiang Province is China’s ecological-civilization construction-demonstration area and a common prosperity-demonstration province. Analyzing the effect of the realization of its forest ecological products’ value and studying the optimization path can help provide a scientific basis for promoting the realization of regional, urban agglomeration, and national ecological products’ value. However, there are still few studies on the evaluation and optimization path of the forest ecological products’ value-realization effect. It is necessary to strengthen research in this aspect.

## 5. Conclusions

Based on the relevant theories and methods of ecological products’ value accounting, this paper constructed an indicators system for GEP accounting, applied the panel data of Zhejiang Province from 2000 to 2020 for analysis, and established a Super-SBM model and the Malmquist index, to set out to measure the value-conversion efficiency of forest ecological products in Zhejiang Province. The following conclusions were obtained:

(1) The value of forest ecological products in Zhejiang Province fluctuated and increased. The value of forest ecological products in 2020 was RMB 973.717 billion. Among them, the value of material products was RMB 12.56 billion, the value of ecological regulatory products was RMB 726.323 billion, and the value of cultural service products was RMB 234.834 billion. Carbon fixation and oxygen release, soil conservation, and cultural service were the main ecological products of forests in Zhejiang Province.
(2) Overall value-conversion efficiency of forest ecological products in Zhejiang Province was generally good, but there were large differences among cities. The value-conversion efficiency of forest ecological products in each city showed an increasing phenomenon, and the cities that were in a state of inefficiency gradually approached the production frontier. It indicates that the overall value-conversion efficiency of forest ecological products in Zhejiang Province improved as a whole.
(3) The total-factor productivity of forest ecological products in Zhejiang Province showed a fluctuating trend, growing rapidly from 2000 to 2010, and declining from 2010 to 2020. Changes in total-factor productivity had a positive relationship with technological progress. However, six cities have initially declined.
(4) The main reason for the loss of conversion efficiency of forest ecological products’ value was excessive input and insufficient output. The specific causes of efficiency loss varied from region to region. Therefore, each city should find a path to improve the conversion efficiency, according to the actual situation.

In the calculation of regulatory ecological products, this paper used the accounting method of ecosystem services, combined with the InVEST model and ArcGIS software for physical-quantity accounting, and then adopted the conversion-efficiency method to calculate the value conversion of the “two mountains” in Zhejiang Province. Traditionally, the value conversion of the “two mountains” has been assessed by measuring the Green Gold Index. This paper chose the conversion-efficiency method to calculate instead, which made up for the shortcomings of the overly simple calculation method and could reflect the key factors affecting the conversion efficiency of forest ecological products’ value, to a certain extent. However, there are some deficiencies in the GEP accounting work, such as strong technicality, more basic data required, and the ununified evaluation methods and model parameters of some service functions, which easily affect the accuracy and stability of the results. It should be noted that the types of forest ecological products accounted for in this study were not comprehensive, due to the difficulty of accounting. This paper only calculated seven forest ecological products’ value indicators (material product value, water retention, nutrient accumulation, carbon fixation and oxygen release, air purification, soil conservation, and cultural service). In future research, it is necessary to further improve the evaluation system of forest ecological products’ value-conversion efficiency.

## Figures and Tables

**Table 1:** Conversion efficiency index system and method of forest ecological products’ value in Zhejiang Province.

Category	Primary Index	Secondary Index	Tertiary Index	Methods
Input	Value of forest ecological products	Material products value	Forest product	Marketing value method
		Regulatory products value	Water retention	Marketing value method
			Nutritional cumulation	Replacement cost
			Carbon fixation and oxygen release	Replacement cost
			Air purification	Replacement cost
			Soil conservation	Shadow-engineering method
		Cultural service	Forest recreation	Travel-cost method
	Labor force	Number of employees in the forestry industry		
	Capital	Sum of investments in forestry fixed assets used		
Output	Output value of the forestry industry	Output value of primary forestry industry		
		Output value of secondary forestry industry		
		Output value of tertiary forestry industry		

**Table 2:** The value of forest ecological products in Zhejiang Province in 2020.

Ecological Products/Services	Magnitude of Value/×10^8^	Proportion/%
Material products	125.60	1.29
Water retention	0.57	0.01
Nutrient accumulation	274.68	2.82
Carbon fixation and oxygen release	4188.58	43.02
Air purification	31.32	0.32
Soil conservation	2768.09	28.42
Cultural service	2348.34	24.12
Sum	9737.17	100

**Table 3:** Value of various ecological products in Zhejiang Province from 2000 to 2020.

Year	Sum	Material Products	Regulatory Products	Water Retention	Nutritional Accumulation	Carbon Fixation and Oxygen Release	Air Purification	Soil Conservation	Cultural Service
2001	6724.69	47.41	6663.44	0.45	277.01	4224.17	26.27	2135.54	13.84
2005	6729.24	45.34	6594.46	0.43	275.40	4199.68	31.69	2087.20	89.44
2010	8138.28	59.66	7854.32	0.29	275.09	4194.86	31.69	3352.40	224.30
2013	10,123.19	56.95	9496.54	0.40	275.94	4207.84	31.34	4981.02	569.70
2015	10,075.43	74.11	9457.30	0.38	273.69	4173.51	28.70	4981.02	544.02
2018	7715.58	111.88	6490.39	0.43	273.66	4173.09	31.32	2011.89	1113.31
2020	9737.17	125.60	7263.23	0.57	274.68	4188.58	31.32	2768.09	2348.34

Note: Sum represents the total value of all ecological products. Unit: RMB 100 million.

**Table 4:** Ranking of the mean value-conversion efficiency of forest ecological products in cities in Zhejiang Province from 2000 to 2020.

DMU	2001	2005	2010	2013	2015	2018	2020	Average	Rank
Hangzhou	0.09	0.21	0.30	1.20	1.09	0.38	0.42	0.52	11
Ningbo	1.27	1.39	1.51	1.06	1.17	1.32	1.08	1.30	5
Wenzhou	0.02	1.02	0.44	0.47	1.26	1.10	1.16	0.78	10
Jiaxing	17.77	3.39	3.54	3.37	3.38	3.33	3.24	5.56	1
Huzhou	1.24	1.57	1.66	1.87	1.56	2.02	2.05	1.71	2
Shaoxing	1.44	1.60	1.43	1.02	1.21	1.06	1.17	1.27	6
Jinhua	1.86	1.47	1.09	1.13	1.34	1.88	1.67	1.39	4
Quzhou	0.28	1.22	1.28	1.05	1.10	1.13	1.25	1.03	9
Taizhou	2.07	1.82	1.39	1.32	1.06	1.09	1.31	1.60	3
Lishui	1.09	1.15	2.61	1.31	1.22	1.21	1.21	1.25	7
Zhoushan	1.16	1.30	1.35	1.25	1.23	1.36	1.02	1.23	8
Average	2.60	1.47	1.51	1.37	1.42	1.44	1.42	—	

**Table 5:** Annual Variation Index and Decomposition Index of Zhejiang Province from 2000 to 2020.

Period	EFFCH	TECH	PECH	SECH	TFP
2001–2005	1.021	0.006	1.13	0.904	0.006
2005–2010	1.071	5.809	1.026	1.043	1.419
2010–2013	0.876	1.005	0.964	0.909	0.881
2013–2015	1.101	0.525	1.022	1.077	0.578
2015–2018	0.792	1.101	0.793	1	0.873
2018–2020	1.317	0.534	1.272	1.036	0.703
Average	1.016	1.024	1.024	0.993	1.04

**Table 6:** Overall change index and decomposition index of value-conversion efficiency of ecological products in cities of Zhejiang Province from 2001 to 2020.

DMU	EFFCH	TECH	PECH	SECH	TFP
Hangzhou	1.207	0.855	1.196	1.009	1.031
Ningbo	1.03	0.982	1.085	0.95	1.012
Wenzhou	1.002	0.885	1	1.002	0.886
Jiaxing	1	0.743	1	1	0.743
Huzhou	0.959	0.79	1	0.959	0.758
Shaoxing	0.95	0.749	1	0.95	0.712
Jinhua	1	1.01	1	1	1.01
Quzhou	1.052	0.864	1	1.052	0.909
Taizhou	1	0.796	1	1	0.796
Lishui	1	5.617	1	1	5.617
Zhoushan	1	1.013	1	1	1.013
Average	1.016	1.024	1.024	0.993	1.04

**Table 7:** Average annual input–output slack rate of cities in Zhejiang Province.

DMU	Redundancy Rate					Insufficient Rate		
	Material Products	Regulatory Products	Cultural Service	Number of Employees in the Forestry Industry	Forestry Investment	Output Value of Primary Forestry Industry	Output Value of Secondary Forestry Industry	Output Value of Tertiary Forestry Industry
Hangzhou	0.33	0.21	0.22	0.31	0.47	0.09	2.33	0.83
Ningbo	0.02	0.05	0.00	0.66	0.27	0.18	0.01	0.01
Wenzhou	0.04	0.15	0.12	15.87	0.01	0.46	14.23	0.20
Jiaxing	1.76	0.43	5.38	1.54	0.00	0.87	0.93	0.00
Huzhou	0.00	0.08	0.60	0.00	0.03	0.00	0.33	0.53
Shaoxing	0.00	0.45	0.10	0.00	0.31	0.22	0.00	0.00
Jinhua	0.70	0.14	0.01	0.02	0.08	0.25	0.00	0.17
Quzhou	0.11	0.48	0.06	0.12	0.00	0.08	0.29	0.00
Taizhou	0.40	0.16	0.00	0.22	0.14	0.09	0.19	0.21
Lishui	0.83	0.96	0.08	0.13	0.10	0.02	0.73	0.09
Zhoushan	0.01	0.00	0.00	0.11	0.00	0.00	0.00	0.53

Note: The redundancy rate is equal to the input-factor slack divided by the corresponding input-index value. The insufficiency rate is equal to the slack in the total-agricultural-output value divided by the corresponding total-agricultural-output value to obtain the agricultural-output value.

## Data Availability

Not applicable.

## References

[1] Ouyang Z.Y., Wang X.K. (1999). A primary study on Chinese terrestrial ecosystem services and their ecological-economic values. Acta Ecol. Sin..

[2] Postel S., Bawa K., Kaufman L., Peterson C.H., Carpenter S., Tillman D., Dayton P., Alexander S., Lagerquist K., Goulder L. (1997). Nature’s Services: Societal Dependence on Natural Ecosystems.

[3] Costanza R., d’Arge R., De Groot R., Farber S., Grasso M., Hannon B., Limburg K., Naeem S., O’neill R.V., Paruelo J. (1997). The value of the world’s ecosystem services and natural capital. Nature.

[4] Bolund P., Hunhammar S. (1999). Ecosystem services in urban areas. Ecol. Econ..

[5] Drakou E.G., Pendleton L., Effron M., Ingram J.C., Teneva L. (2017). When ecosystems and their services are not co-located: Oceans and coasts. ICES J. Mar. Sci..

[6] Makwinja R., Kaunda E., Mengistou S., Alamirew T. (2021). Impact of land use/land cover dynamics on ecosystem service value—a case from Lake Malombe, Southern Malawi. Environ. Monit. Assess..

[7] Dardonville M., Legrand B., Clivot H., Bernardin C., Bockstaller C., Therond O. (2022). Assessment of ecosystem services and natural capital dynamics in agroecosystems. Ecosyst. Serv..

[8] Hu X.J., Song C.S., Fan X.Y., Xiao Y., Xu W.H., Ouyang Z.Y. (2021). Main Regulation Services and Value Assessment of Shrub Ecosystem in China. J. Beijing For. Univ..

[9] Shao M., Ma L.P., Wang X.Y., Che X.H., Wang F., Lu J.F., Luo W.Y. (2022). The valuation of ecosystem service value of desertification grassland from 2004 to 2014 in Hexi Corridor, China. J. Desert Res..

[10] Zeng X.G., Yu H.Y., Xie F. (2014). Concept, Classification and Market Supply Mechanism of Ecological Products. China Popul. Resour. Environ..

[11] Huang R.L. (2015). Study on the Ecological Product Value Assessment. China Population. Resour. Environ..

[12] Liu J.Y., Mou D.G. (2020). Research Progress of Ecological Product Value and Its Realization Mechanism. Ecol. Econ..

[13] Lu X.C. (2022). Research on the Value Realization Path of Beijing Ecological Products in the New Development Stage. Ecol. Econ..

[14] Yu G.R., Yang M. (2022). Ecological economics foundation research on ecological values, ecological asset management, and value realization: Scientifical concepts, basic theories, and realization paths. Chin. J. Appl. Ecol..

[15] Li Y., Cheng S.L., Huang J., Fu X. (2021). The Present Situation and Prospect of Research on Ecological Product Value Realization——Based on Bibliometric Analysis. For. Econ..

[16] Qiu S.L., Jin L.S. (2019). International Experience and Reference of Value Realization of Ecological Goods. Reform Econ. Syst..

[17] Tan R. (2021). Value Realization of Ecological Products and Innovation of Governance Mechanism. Chin. Land.

[18] Song C.S., Ouyang Z.Y. (2020). Gross Ecosystem Product accounting for ecological benefits assessment: A case study of Qinghai Province. Acta Ecol. Sin..

[19] Dolkar P., Xiao Y., Ouyang Z.Y., Wang L.Y. (2020). Assessment of ecological conservation effect in Xishui county based on gross ecosystem product. Acta Ecol. Sin..

[20] Chen Z.Z., Lei J.R., Wu T.T., Chen D.X., Zhou Z., Li Y.L., Hong X.J., Yang Z.Y., Li Y.D. (2021). Gross ecosystem product accounting of national park: Taking Hainan Tropical Rainforest National Park as an example. Chin. J. Appl. Ecol..

[21] Fan Z.L., Li W.M. (2020). Research on the Realization Mechanism of Ecological Product Value-A case Study of Guizhou Province. J. Hebei Geo Univ..

[22] Hao L.H., Chen S., He S. (2021). Accounting Method and Application on the Marine Provisioning Ecological Product Values: Taking Wenzhou City’s Practice as an Example. Environ. Prot..

[23] Lin Y.Y., Xu X.B. (2022). Spatiotemporal variations of gross ecosystem product and identification of important ecological protection spaces in the Yangtze River Delta. Resour. Sci..

[24] Wu Z.J., Du S.M., Huang Y., Zheng B.Y., Xie Z.Y., Luo C.K., Wang F., Zhu J.Q. (2022). Assessment of ecological conservation effect in southern Jiangxi Province based on gross ecosystem product. Acta Ecol. Sin..

[25] Cheng C.Y., Ge C.Z., Du Y.C., Li J.D. (2019). Green Gold Index accounting for Quzhou City in Zhejiang Province. Acta Ecol. Sin..

[26] Ma G.X., Yu F., Wang J.N., Zhou X.F., Yuan J., Mou X.J., Zhou Y., Yang W.S., Peng F. (2017). Measuring gross ecosystem product (GEP) of 2015 for terrestrial ecosystems in China. China Environ. Sci..

[27] Chen M., Ji R.T., Liu X., Liu C.W., Su L.H., Zhang L.J. (2021). Gross ecosystem product accounting for ‘Two Mountains’ Bases and transformation analysis: The case study of Ninghai County. Acta Ecol. Sin..

[28] Krieger D.J. (2001). Economic Value of Forest Ecosystem Services: A Review. Wilderness Soc..

[29] Ferraro P.J., Lawlor K., Mullan K.L., Pattanayak S.K. (2012). Forest Figures: Ecosystem Services Valuation and Policy Evaluation in Developing Countries. Rev. Environ. Econ. Policy.

[30] Xu T.Y., Niu X., Wang B. (2021). Spatial patterns of forest ecosystem services in Liaoning province. Sci. Soil Water Conserv..

[31] Chen G.H., Jin L., Zhan J.W., Chen R.N., Sun L.J., Ren Y.D., Yang Y.H. (2021). Analysis on forest ecosystem services and its impact factors in Qingyuan County. Liaoning For. Sci. Technol..

[32] Xie G., Li W., Xiao Y., Zhang B., Lu C., An K., Wang J., Xu K., Wang J. (2010). Forest ecosystem services and their values in Beijing. Chin. Geogr. Sci..

[33] Aznar-Sánchez J.A., Belmonte-Ureña L.J., López-Serrano M.J., Velasco-Muñoz J.F. (2018). Forest Ecosystem Services: An Analysis of Worldwide Research. Forests.

[34] Yu L.Y., Shi T., Guo J.J. (2019). Developing Mechanisms to Realize the Value of Forest Ecological Products. For. Resour. Manag..

[35] Luo J., Li L., Jiang Y., Zhou X.L. (2022). Evaluation of service function of forest ecosystem in Hunan province. Ecol. Sci..

[36] Zuo S.L., Jin S.S., Gu X.Q. (2022). Assessment of forest ecosystem service value accounting in priority area of biodiversity protection in Anji county of geography census. Bull. Surv. Mapp..

[37] Mavi N.K., Mavi R.K. (2019). Energy and environmental efficiency of OECD countries in the context of the circular economy: Common weight analysis for Malmquist productivity index. J. Environ. Manag..

[38] Zhang Z., Liao X.P., Li C.H., Yang C., Yang S.S., Li Y.H. (2022). Spatio-temporal Characteristics of Agricultural Eco-efficiency and Its Determinants in Hunan Province. Econ. Geogr..

[39] Andrejic M., Kilibarda M., Pajic V. (2021). Measuring efficiency change in time applying malmquist productivity index: A case of distribution centres in Serbia. Facta Univ. Ser. Mech. Eng..

[40] Yang X., Wang W., Wu H., Wang J., Ran Q., Ren S. (2021). The impact of the new energy demonstration city policy on the green total factor productivity of resource-based cities: Empirical evidence from a quasi-natural experiment in China. J. Environ. Plan. Manag..

[41] Jin C., Lu Y.Q. (2021). Review and Prospect of Research on Value Realization of Ecological Products in China. Econ. Geogr..

[42] Sun B.W., Peng X.S. (2021). Realization Mode, Key Issues and Institutional Guarantee System of Ecological Product Value. Ecol. Econ..

[43] Liu Y.X., Shi X.L., Shi W.J. (2021). Evaluation of water retention services of forest ecosystems in Fujian Province: Comparison between results from the InVEST model and meta-analysis. Acta Ecol. Sin..

[44] Ma Z.D., Gao H., Yang J., Xi J.C., Li X.M., Ge Q.S. (2014). Valuation of Nansihu Lake Wetland Ecosystem Services Based on Multi-Sources Data Fusion. Resour. Sci..

[45] Wang L., Xiao Y., Ouyang Z.Y., Wei Q., Bo W., Zhang J., Ren L. (2017). Gross ecosystem product accounting in the national key ecological function area. China Popul. Resour. Environ..

[46] Tone K. (2002). A slacks-based measure of super-efficiency in data envelopment analysis. Eur. J. Oper. Res..

[47] Wang S.J., Gao S., Huang Y.Y., Shi C.Y. (2020). Spatio-temporal evolution and trend prediction of urban carbon emission performance in China based on super-efficiency SBM model. Acta Ecol. Sin..

[48] Sun Y.X., Cheng Y., Liu N. (2021). Spatiotemporal evolution of China’s high quality economic development and its driving mechanism of scientific and technological innovation. Resour. Sci..

[49] Li J.J., Jing Y.J. (2021). Research on Environmental Efficiency Measurement and Influencing Factors Based on DEA-Malmquist-Tobit Model: Taking Henan Province as an Example. Ecol. Econ..

[50] Bjurek H. (1996). The Malmquist Total Factor Productivity Index. Scand. J. Econ..

[51] Chen Z., Zhang X. (2000). Value of ecosystem services in China. Chin. Sci. Bull..

[52] Qing L., Chun D., Dagestani A.A., Li P. (2022). Does Proactive Green Technology Innovation Improve Financial Performance? Evidence from Listed Companies with Semiconductor Concepts Stock in China. Sustainability.

[53] Qing L., Chun D., Ock Y.S., Dagestani A.A., Ma X. (2022). What Myths about Green Technology Innovation and Financial Performance’s Relationship? A Bibliometric Analysis Revie. Economies.

[54] Zhang Y.N., Zhang L.T., Han L.M. (2021). Study on the Evaluation and Promotion Path of Agricultural Ecological Efficiency: An Empirical Analysis of 17 Prefecture Level Cities in Shandong Province. Ecol. Econ..

